# A single-amino-acid in-frame deletion in CYP17A1 results in combined 17-hydroxylase and 17,20-lyase deficiency in an Iranian family despite the protein mutation site

**DOI:** 10.1038/s41439-021-00160-y

**Published:** 2021-07-21

**Authors:** Ashkan Habib, Alireza Shojazadeh, Mohadeseh Molayemat, Hossein Jafari Khamirani, Sina Zoghi, Seyed Alireza Dastgheib, Asadollah Habib

**Affiliations:** 1 School of Medicine, Shiraz University of Medical Sciences, Shiraz, Iran; 2 Department of Medical Genetics, Shiraz University of Medical Sciences, Shiraz, Iran; 3 Comprehensive Medical Genetic Center, Shiraz University of Medical Sciences, Shiraz, Iran; 4 Student Research Committee, Shiraz University of Medical Sciences, Shiraz, Iran; 5 Department of Endocrinology, School of Medicine, Kazerun Branch, Islamic Azad University, Kazerun, Iran

**Keywords:** Next-generation sequencing, Adrenal gland diseases, Hypogonadism

## Abstract

In this study, we detected homozygous mutations in the CYP17A1 gene (NM_000102.4:c.1053_1055delCCT; p.Leu353del; SCV001479329) in a 28-year-old female patient (46,XX) and her phenotypically female 30-year-old sister (46,XY) who had phenotypes consistent with combined 17-hydroxylase and 17,20-lyase deficiency. The phenotypes were not expected based on the location of the mutation in the CYP17A1 redox partner-binding site and a previous description of the same mutation linked with isolated 17,20-lyase deficiency.

Congenital adrenal hyperplasia (CAH) is a group of disorders caused by deficiencies in enzymes that are involved in the synthesis of cortisol and sex steroid hormones due to genetic mutations. Deficiencies in 17-hydroxylase and 17,20-lyase, with an incidence of 1:1,000,000 births, are the rarest known types of CAH^1^. The gene encoding the CYP17A1 protein, which has both 17-hydroxylase and 17,20-lyase activities, contains eight exons and is located on 10q24.3^2^. Defects in CYP17A1 reduce sex steroid and cortisol levels, which leads to increases in adrenocorticotropic hormone (ACTH) secretion as well as progesterone and 11-deoxycorticostrone accumulation. Patients with this type of deficiency develop female genitalia at birth (46, XX, or XY), show an absence of secondary sexual characteristic development at puberty, and exhibit amenorrhea, hypertension, hypokalemia, and suppression of the renin–angiotensin–aldosterone system. However, it should be noted that 10–15% of these patients are normotensive when diagnosed^3^. More than 140 disease-causing variants in CYP17A1 have been reported^4^. Mutations affecting different regions of CYP17A1 can lead to varying degrees of severity of 17-hydroxylase deficiency. While mutations in the heme-binding (p.R440C), substrate-binding (D487_F489del), and steroid-binding sites (p.H373L) of CYP17A1 usually result in combined 17-hydroxylase/17,20-lyase deficiency, mutations in the redox partner-binding site (p.E305G) lead to isolated 17,20-lyase deficiency (ILD)^3^. In this study, we present a southern Iranian family whose symptoms and laboratory tests are consistent with combined 17-hydroxylase/17,20-lyase deficiency but are unexpected based on the location of the mutation in the redox partner-binding site and a previous description of the same mutation linked with ILD.

The proband (IV6; Fig. 1a) was a 28-year-old phenotypic female who initially presented with repeated episodes of paralysis beginning at the age of 18 years. The patient’s condition was first diagnosed as a consequence of hypokalemia (hypokalemic periodic paralysis), for which spironolactone was prescribed. Additionally, she had shown a generally darker skin color than the other members of her family since childhood. She had phenotypically female external genitalia with no signs of secondary sexual characteristics, including the absence of menarche, breast development, and pubic/axillary hair (Tanner stage B1P1). Her height was measured as 171 cm, which was taller than the heights of the rest of her family, and her weight was 73 kg. Her blood pressure was 130/80 mmHg, and abdominal ultrasonography revealed a hypoplastic uterus and ovaries.

**Fig. 1:**
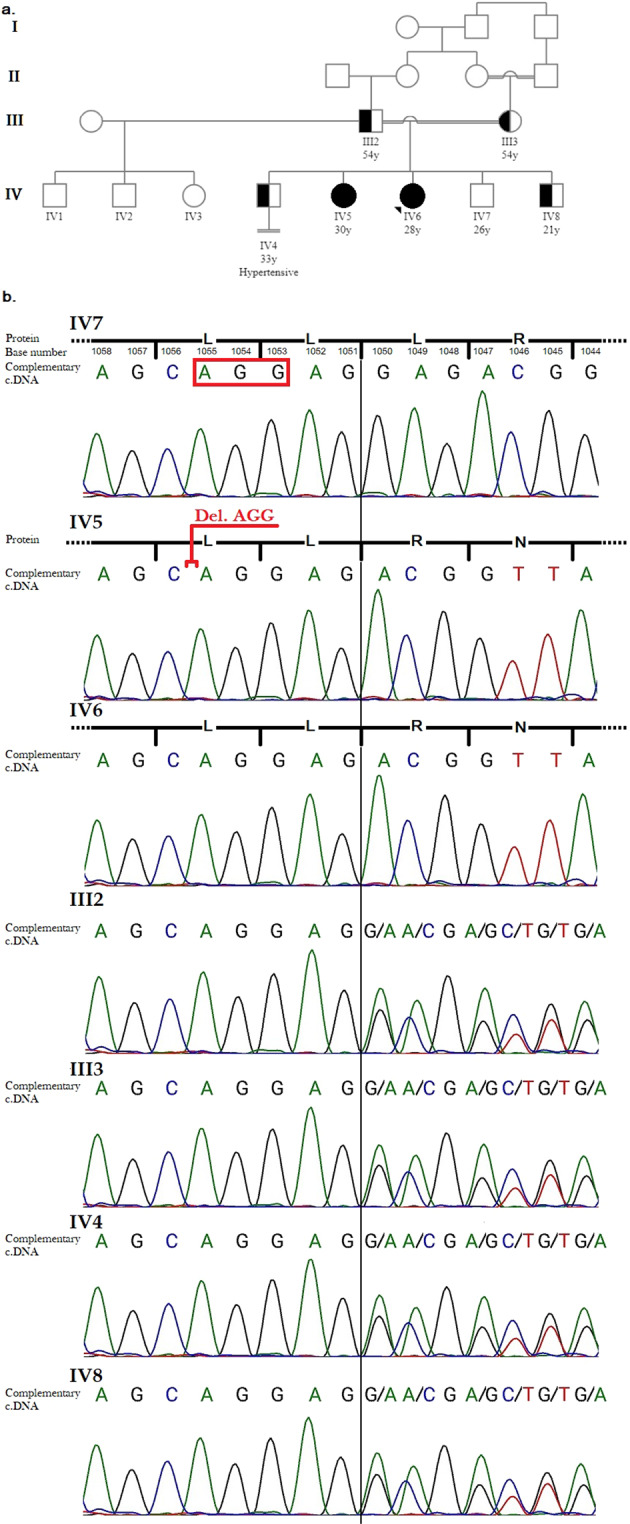
The pedigree and the electropherogram for the proband and her family. **a** The pedigree of the family. Circles and squares represent females and males, respectively. Open, half-solid and solid shapes indicate wild-type, heterozygous or homozygous status for the mutation, respectively. Marriages indicated with double lines are consanguineous. The arrow represents the proband. **b** Electropherogram of the complement sequence for the proband and her family. c.1053_1055 is located at a position that crosses two leucine codons (codons 351 and 352). In addition, leucine repeats occur from 350Leu_353Leu. However, in accordance with HGVS rules, the mutation name is determined as the deletion of 353Leu.

She was born from consanguine parents (first-degree cousins; Fig. 1a). Her family included two daughters and three sons. The proband’s 30-year-old sister (IV5) had very similar symptoms, which are discussed below. Her 33-year-old married brother (IV4) was hypertensive and had been infertile for almost 10 years, since the time of his marriage, despite showing normal sperm analysis, scrotal ultrasonography, hormone profile, and karyotype (46 XY) results. The proband’s 26-year-old married (IV7) and 21-year-old single (IV8) brothers were both symptom-free at the time of this study and had no issues related to the subject of this study.

The proband’s 30-year-old sister (IV5) also had shown a darker skin color since childhood and had phenotypically female external genitalia with no signs of secondary sexual characteristics, including the absence of breast development, pubic/axillary hair (Tanner stage B1P1), and menstrual periods. However, unlike the proband, she had no history of paralysis episodes. Karyotyping revealed a 46 XY karyotype, the presence of undescended intra-abdominal testes was therefore presumed. At the age of 27 years, bilateral orchiectomy was performed on the patient. In a physical examination, her height was measured as 179 cm, and her weight was 65.7 kg. She was hypertensive, with a blood pressure of 140/90 mmHg.

Written informed consent was obtained from each subject. The study was conducted based on principles of the Declaration of Helsinki, and the study protocol was approved by the Institutional Review Board of Shiraz University of Medical Sciences. Genomic DNA was extracted from peripheral white blood cells using a QIAamp DNA Blood Mini Kit. Whole-exome sequencing (WES) was performed on the DNA of the proband using a HiSeq 3000/4000 SBS Kit.

The raw data were converted by HiSeq X and then aligned against the human reference genome (hg19) by using Burrows–Wheeler Aligner. The core phenotypes associated with the identified mutations were obtained from the OMIM database (OMIM #202110).

We confirmed the presence of the mutation identified in this study by Sanger sequencing. PCR primers were designed using Oligo Primer Designer.

A mutation (NM_000102.4:c.1053_1055delCCT; p.Leu353del; SCV001479329) located in exon 6 of the CYP17A1 gene was detected in the proband. Although this variant was not listed in the gnomAD browser beta (https://gnomad.broadinstitute.org/) and ClinVar (https://www.ncbi.nlm.nih.gov/clinvar/) databases, it was previously reported with extensively different phenotype by Hahm et al. when occurring in compound heterozygosity^5^ and by Nazari et al. when occurring in homozygosity^6^. The proband and her sister (IV5 and IV6) were recognized as homozygous for the disease-causing variant. All other family members except for IV7 were heterozygous for the variant. The presence of the variant was confirmed by Sanger sequencing (Fig. 1b). The variant was classified as likely pathogenic based on ACMG-AMP guidelines (PM2, PM4, PP3, PP4)^7^.

The karyotype of the proband was 46,XX, with an inversion in ch.9 (p.11, q.13), and that of her older sister was 46,XY. Hypokalemia, borderline low renin levels, high ACTH levels, and decreased 17 hydroxyprogesterone, estradiol, testosterone, androstenedione, dehydroepiandrosterone sulfate (DHEA-S), and cortisol levels were observed in the proband and her older sister. All other family members had normal laboratory results (Table S1).

Figure 2 illustrates the location of the deleted amino acid in the protein structure, which is within both the K helix and the redox partner binding site. The deleted amino acid shares a noncovalent bond with 331Glu in the J helix (Fig. 2).

**Fig. 2:**
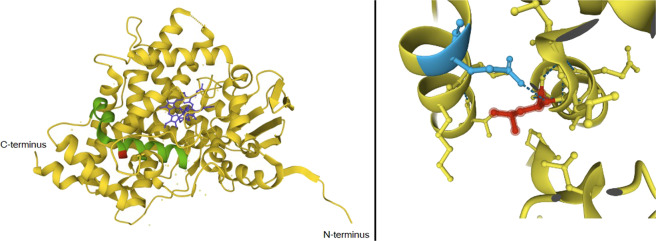
3D illustration of the CYP17A1 protein. The locations of heme and steroids (blue), the K helix (green), and the p.L353del (red) are shown on the left. The deleted leucine amino acid (red) in the K helix and its noncovalent bond with 331Glu (blue) in the J helix are shown on the right.

The mutation was previously reported to be homozygous in an Iranian patient by Nazari et al.^6^. However, the authors reported that the patient showed a normal cortisol level, elevated 17-hydroxysteroid level, higher but still insufficient estradiol level, and ambiguous genitalia, indicating the existence of some 17-hydroxylase activity and, thus, resulting in a diagnosis of ILD (Table S2). Indeed, mutations located near 353Leu, including R347H, R347C, and R358Q, result in ILD with normal cortisol and 17OH-progesterone levels, usually ambiguous genitalia, and normal blood pressure and electrolytes, as they are located in the redox partner binding site. Some patients with such mutations also develop pubic hair and gynecomastia (Table S2). In these mutations, some 17-hydroxylyase activity is retained^8^. These findings indicate that the phenotype resulting from p.Leu353del reported in our study results in combined 17-hydroxylase/17,20-lyase deficiency, which is unexpected based on both the location of the mutation and the ILD diagnosis reported in a previous study.

## HGV database

The relevant data from this Data Report are hosted at the Human Genome Variation Database at 10.6084/m9.figshare.hgv.3072.

## Supplementary information

Table 1

Table 2
