# Supplementary material for: A single-amino-acid in-frame deletion in CYP17A1 results in combined 17-hydroxylase and 17,20-lyase deficiency in an Iranian family despite the protein mutation site
Source: Hum Genome Var. 2021 Jul 21;8:31. doi: 10.1038/s41439-021-00160-y (PMC8295247; doi:10.1038/s41439-021-00160-y)
Supplement: Supplementary file 1 — Table 1 [file 41439_2021_160_MOESM1_ESM.docx]

| **Table 1. Clinical lab results for the subjects in the study.** | | | | | | | | |
| --- | --- | --- | --- | --- | --- | --- | --- | --- |
|  | Proband (IV6) | Proband Sister (IV5) | Proband Oldest Brother (IV4) | Proband Middle Brother (IV7) | Proband Youngest Brother (IV8) | Proband Father (III2) | Proband Mother (III3) | Reference Range |
| Age (years) | 28 | 30 | 33 | 26 | 21 | 54 | 54 |  |
| WBC (x1000/µL) | 6.1 | 9.1 | 6.8 | 8.7 | 6.9 | 6.2 | 8.2 | 4.5-11 |
| Hb (g/dL) | 12.1 | 14.2 | 13.7 | 13.9 | 14.6 | 13.7 | 13.7 | Men:13.5-18.0  Women:12-16 |
| MCV (fL) | 79.37 | 89.19 | 90.32 | 77.16 | 92.69 | 85.59 | 85.43 | 80-100 |
| Plt (x1000/µL) | 292 | 340 | 210 | 199 | 210 | 219 | 242 | 150-450 |
| FBS (mg/dL) | 98 | 99 | 98 | 102 | 88 | 111 | 111 | 70-99 |
| BUN (mg/dL) | 10 | 9 | 12 | 11 | 16 | 22 | 18 | 5-23 |
| Creatinine (mg/dL) | 0.7 | 0.7 | 0.9 | 0.8 | 0.9 | 0.9 | 0.8 | 0.6-1.4 |
| Sodium (meq/L) | 137 | 138 | 140 | 142 | 141 | 141 | 141 | 133-146 |
| Potassium (meq/L) | 3.7 | 4.1 | 4.1 | 4.1 | 4.2 | 4.1 | 4.3 | 3.5-5.5 |
| Chloride (mMol/L) | 108 | 110 | 108 | 107 | 109 | 106 | 111 | 98-109 |
| TSH (µIU/mL) | 2.1 | 0.96 | 0.98 | 3.21 | 1.96 | 0.78 | 0.44 | 0.27-5.5 |
| FT4 (Pmol/L) | 14.9 | 15.3 | 15.6 | 16.6 | 16.3 | 13.62 | 15.86 | 12-22 |
| LH (mIU/mL) | 51.2 | 44.3 | 3.14 | 4.21 | 3.68 | 4.35 | 28.83 | Follicular: 1.5-8.0  Ovulation: 9.6-80.0  Luteal:0.2-6.5  Post-menopausal:8.0-33.0  Men: 1.1-7.0 |
| FSH (mIU/mL) | 40.3 | 65.1 | 4.11 | 3.69 | 5.17 | 4.5 | 83.08 | Follicular: 2.9-12.0  Ovulation: 6.3-24  Luteal:1.5-7.0  Post-menopausal:17-95  Men: 1.7-12 |
| DHEA-SO4 (µg/mL) | 0.12 | 0.11 | 1.63 | 1.12 | 0.96 | 0.62 | 0.58 | Men:0.06-4.58  Women:0.03-5.88 |
| Androstenedione (ng/mL) | 1.01 | 0.83 | 2.39 | 1.66 | 2.32 | 2.36 | 1.22 | Follicular:0.75-3.1  Luteal:0.94-3.2  Men:0.6-2.7 |
| Cortisol (µg/dL) | 3.21 | 3.31 | 9.63 | 9.63 | 6.19 | 11.41 | 14.9 | Morning: 5-23 |
| ACTH 8 AM (pg/mL) | 79.2 | 97.2 | 33.8 | 28.7 | 32.6 | 36.99 | 46.74 | 7.2-63.3 |
| 17-OH Progesterone (ng/mL) | 0.08 | 0.06 | 1.89 | 2.68 | 1.89 | 1.53 | 0.51 | Follicular:0.05-1.02  Luteal:0.3-2.34  Post-menopausal:<0.93  Men:0.31-2.01 |
| Testosterone (ng/mL) | <0.025 | <0.025 | 2.93 | 5.21 | 4.21 | 4.15 | 0.19 | Men:2.3-8.58  Women:0.1-0.9 |
| Estradiol (pg/ml) | <5 | <5 | 18.39 | 20.01 | 22.21 | 14.32 | <5 | Follicular: 18-147  Pre-Ovulation: 93-575  Luteal:43-214  Post-menopausal:<58  Men:5-62 |
| Aldosterone (pg/mL) | 88.3 | 159.2 | 154.9 | 132.5 | 143.6 | 136.7 | 144.1 | 30-400 |
| Plasma renin (µIU/mL) | 7.2 | 8.6 | 37.1 | 30.4 | 26.4 | 33.0 | 25.2 | 4.4-46.1 |
| AMH (ng/mL) | 0.42 | 0.09 | N/A | N/A | N/A | N/A | N/A | 0-12.6 |
| Note: Samples were taken in the upright position; The increased levels of LH and FSH and the decreased estradiol in the mother of siblings is due to menopause.  Abbreviations: WBC, white blood cell; Hb, hemoglobin; MCV, mean corpuscular volume; Plt, platelet; FBS, fasting blood sugar; BUN, blood urea nitrogen; TSH, thyroid stimulating hormone; FT4, free T4; LH, luteinizing hormone; FSH, follicle stimulating hormone; DHEA-SO4, Dehydroepiandrosterone sulfate; ACTH, Adrenocorticotropic hormone; AMH, Anti-Müllerian hormone; N/A, not applicable. | | | | | | | | |
