# Supplementary material for: A single-amino-acid in-frame deletion in CYP17A1 results in combined 17-hydroxylase and 17,20-lyase deficiency in an Iranian family despite the protein mutation site
Source: Hum Genome Var. 2021 Jul 21;8:31. doi: 10.1038/s41439-021-00160-y (PMC8295247; doi:10.1038/s41439-021-00160-y)
Supplement: Supplementary file 2 — Table 2 [file 41439_2021_160_MOESM2_ESM.docx]

| **Table 2. Comparison of symptoms in studies with mutation in CYP17A1 redox partner-binding site.** | | | | | | | | | | |
| --- | --- | --- | --- | --- | --- | --- | --- | --- | --- | --- |
| Study | Habib et al. | | Nazari et al.^1^ | Hahm et al.^2^ | Geller et al.^3^ | | Van Den Akker et al.^4^ | | | |
| Genetic State | HM | HM | HM | CHZ | HM | HM | CHZ | CHZ | HM | HM |
| Mutation | p.L353del^a^ | p.L353del^a^ | p.L351del^a^ | p.L350del^a^, p.Y329KfsX90 | p.R347H | p.R.358Q | p.R347C, p.G69AfsX26 | p.R347C, p.P480HfsX327 | p.R347H | p.R347H |
| Diagnosis | CHD | CHD | ILD | CHD | ILD | ILD | ILD | ILD | ILD | ILD |
| Age | 28 years | 30 years | 14 years | 32 years | 13 months | 16 years | 10 years | 28 years | 7.5 years | 2.5 years |
| Karyotype | 46, XX | 46, XY | 46, XX | 46, XX | 46, XY | 46, XY | 46, XY | 46, XY | 46, XY | 46, XY |
| Genitalia | Female | Female | Ambiguous* | Female | Ambiguous* | Ambiguous* | Female | Female | Ambiguous* | Ambiguous* |
| Breast Development | Absent | Absent | Absent | Present* | N/A | Present* | N/A | Absent | N/A | N/A |
| Pubic Hair | Absent | Absent | Absent | Absent | N/A | Present* | N/A | Absent | N/A | N/A |
| Blood Pressure | Elevated | Elevated | Elevated | Elevated | Normal* | Normal* | Elevated | Elevated | (Not Reported) | (Not Reported) |
| Cortisol | Deficient | Deficient | Normal* | Deficient | Normal* | Normal* | Normal* | Normal* | Normal* | Normal* |
| 17OH-progesteron | Borderline low | Borderline low | Elevated*^b^ | Normal* | Normal* | Elevated* | Elevated* | Normal* | Normal* | Normal* |
| * Suggestive of isolated 17, 20-lyase deficiency and partially present 17-hydroxylase activity.  ^a^ Equivalent mutations.  ^b^ Described in the main text, however, exact data not provided.  Abbreviations: HM, homozygous; CHZ, compound heterozygous; CHD, combined 17-hydroxylase/17, 20-lyase deficiency; ILD, isolated 17,20-lyase deficiency; N/A. not applicable.   1. A novel mutation in CYP17A1 gene leads to congenital adrenal hyperplasia: A case report. Nazari M, Yahya Vahidi Mehrjardi M, Neghab N, et al. Int J Reprod Biomed. 2019;17(6):449-454. 2. Hahm JR, Kim DR, Jeong DK, et al. A novel compound heterozygous mutation in the CYP17 (P450 17alpha-hydroxylase) gene leading to 17alpha-hydroxylase/17,20-lyase deficiency. Metabolism. 2003;52(4):488-492. 3. Geller DH, Auchus RJ, Mendonça BB, Miller WL. The genetic and functional basis of isolated 17,20-lyase deficiency. Nat Genet. 1997;17(2):201-205. 4. Van Den Akker ELT, Koper JW, Boehmer ALM, et al. Differential inhibition of 17alpha-hydroxylase and 17,20-lyase activities by three novel missense CYP17 mutations identified in patients with P450c17 deficiency. J Clin Endocrinol Metab. 2002;87(12):5714-5721. | | | | | | | | | | |
